# Supplementary material for: Reversible promoter methylation determines fluctuating expression of acute phase proteins
Source: eLife. 2020 Mar 30;9:e51317. doi: 10.7554/eLife.51317 (PMC7136028; doi:10.7554/eLife.51317)
Supplement: Supplementary file 1. [file elife-51317-supp1.docx]

| **Key Resources Table** | | | | |
| --- | --- | --- | --- | --- |
| **Reagent type (species) or resource** | **Designation** | **Source or reference** | **Identifiers** | **Additional information** |
| gene (*Homo-sapiens*) | *CRP* | NCBI Database | Gene ID：1401 |  |
| gene (*Homo-sapiens*) | *SAA* | NCBI Database | Gene ID: 6288 |  |
| gene (*Homo-sapiens*) | *SAP* | NCBI Database | Gene ID: 325 |  |
| gene (*Homo-sapiens*) | *TET2* | NCBI Database | Gene ID: 54790 |  |
| gene (*Homo-sapiens*) | *DNMT3A* | NCBI Database | Gene ID: 1788 |  |
| cell line (*Homo-sapiens*) | Hep3B | ATCC | ATCC HB-8064 |  |
| transfected construct (human) | pcDNA6.2-GW/EmGFP-miR vector | Invitrogen | catalog number: K4935-00 |  |
| transfected construct (human) | PGL4.10 (luc2) vector | Promega | catalog number: E6651 |  |
| transfected construct (human) | phRL-TK | Promega | catalog number: E6241 |  |
| transfected construct (human) | pcDNA3.1 vector | Invitrogen | catalog number: V795-20 |  |
| transfection reagent | ViaFect | Promega | catalog number: E4982 |  |
| transfection reagent | X-tremeGENE 9 DNA | Roche | catalog number: 06365787001 |  |
| biological sample (human) | Frozen tumor/normal tissue sample | Gansu Cancer hospital |  |  |
| antibody | anti-STAT3  (rabbit polyclonal) | Santa Cruz | catalog number: sc-482X; lot number: B0615 | CHIP: 100:1 |
| antibody | anti-p50  (rabbit polyclonal) | Santa Cruz | catalog number: sc-7178X; lot number: C0314 | CHIP: 100:1 |
| antibody | anti-C/EBP-β  (rabbit polyclonal) | Santa Cruz | catalog number: sc-150X; lot number: J2215 | CHIP: 100:1 |
| antibody | Anti-Dnmt3A  (rabbit monoclonal) | abcam | ab232391 | WB: 1:2000 |
| antibody | Anti-Tet2  (mouse monoclonal) | abcam | ab243323 | WB: 1:1000 |
| recombinant DNA reagent | pKLV-U6gRNA(BbsI)-PGKpuro2ABFP vector (plasmid) | Addgene | catalog number: 50946 |  |
| recombinant DNA reagent | phU6-gRNA vector (plasmid) | Addgene | catalog number: 53188 |  |
| recombinant DNA reagent | pSpCas9(BB)-2A-Puro (PX459) v2.0 vector (plasmid) | Addgene | catalog number: 62988 |  |
| recombinant DNA reagent | pcDNA-*dCas9*-p300 | Addgene | catalog number: 61357 |  |
| sequence-based reagent | human *CRP*-F | This paper | PCR primers (methylation) | GTAGGTGTTGGAGAGGTAGTTATTA |
| sequence-based reagent | human *CRP*-R | This paper | PCR primers (methylation) | ATTTATATCCAAAACAATAAAAAAATTTAC |
| sequence-based reagent | rabbit *CRP*-F | This paper | PCR primers (methylation) | ATGTTAGAGTTGAAGGTGTTGGAGATA |
| sequence-based reagent | rabbit *CRP*-R | This paper | PCR primers (methylation) | AAATACTAAAAATCCTACATCCCTTACCTC |
| sequence-based reagent | human *SAA*-F | This paper | PCR primers (methylation) | GTTTTTATTTTATATTTTTTAGTAG |
| sequence-based reagent | human *SAA*-R | This paper | PCR primers (methylation) | TAATACTAATCTATACTATAACTAAACTAC |
| sequence-based reagent | human *SAP*-F | This paper | PCR primers (methylation) | AAGAAAGAAAAGGTTTTGTTTTTA |
| sequence-based reagent | human *SAP*-R | This paper | PCR primers (methylation) | ATTTTCCAAATCTACCTCCTAAC |
| sequence-based reagent | human *CRP*-F | This paper | PCR primers  (expression) | GGAGCAGGATTCCTTCGGT |
| sequence-based reagent | human *CRP*-R | This paper | PCR primers  (expression) | CACTTCGCCTTGCACTTCAT |
| sequence-based reagent | human *SAA*-F | This paper | PCR primers  (expression) | GTGATCAGCGATGCCAGAGAGA |
| sequence-based reagent | human *SAA*-R | This paper | PCR primers  (expression) | CCAGCAGGTCGGAAGTGATTG |
| sequence-based reagent | human *SAP*-F | This paper | PCR primers  (expression) | CTTGATCACACCGCTGGAGAAG |
| sequence-based reagent | human *SAP*-R | This paper | PCR primers  (expression) | CTTGGGTATTGTAGGAGAAGAGGCTG |
| sequence-based reagent | human *ACTB*-F | This paper | PCR primers  (expression) | CGTGGACATCCGCAAAGAC |
| sequence-based reagent | Human *ACTB*-R | This paper | PCR primers  (expression) | CTCAGGAGGAGCAATGATCTTGA |
| sequence-based reagent | *DNMT1*-F | This paper | miRNA targeting sequences | GATTTGGAAAGAGACAGCTTA |
| sequence-based reagent | *DNMT1*-R | This paper | miRNA targeting sequences | CAACAGAGGACAACAAGTTCA |
| sequence-based reagent | *DNMT3A*-F | This paper | miRNA targeting sequences | GGTGTGTGTTGAGAAGCTGAT |
| sequence-based reagent | *DNMT3A*-R | This paper | miRNA targeting sequences | GAATTTGACCCTCCAAAGGTT |
| sequence-based reagent | *DNMT3B*-F | This paper | miRNA targeting sequences | GGTTTGGCGATGGCAAGTTCT |
| sequence-based reagent | *DNMT3B*-R | This paper | miRNA targeting sequences | CGAGAACAAATGGCTTCAGAT |
| sequence-based reagent | *TET1*-F | This paper | miRNA targeting sequences | CATGCAAGGCCTTCCAGATTA |
| sequence-based reagent | *TET1*-R | This paper | miRNA targeting sequences | AGAGAACAGCCAGTTTGCTTA |
| sequence-based reagent | *TET2*-F | This paper | miRNA targeting sequences | GTGTAGGTAAGTGCCAGAAAT |
| sequence-based reagent | *TET2*-R | This paper | miRNA targeting sequences | CATGGCGTTTATCCAGAATTA |
| sequence-based reagent | *TET3*-F | This paper | miRNA targeting sequences | CCTTTATGACTTCCCTCAGCG |
| sequence-based reagent | *TET3*-R | This paper | miRNA targeting sequences | CCAGTTGATGGACCTGTTCCA |
| sequence-based reagent | *DNMT3A*-F | This paper | Cas9 targeting sequences | GGACCTCTTGGTGGGGCCGG |
| sequence-based reagent | *DNMT3A*-R | This paper | Cas9 targeting sequences | GGAAGGTGGGGCGGCCTGGG |
| sequence-based reagent | *TET2*-F | This paper | Cas9 targeting sequences | GGGAGATGTGAACTCTGGGA |
| sequence-based reagent | *TET2*-R | This paper | Cas9 targeting sequences | GGAGAACTTGCGCCTGTCAG |
| sequence-based reagent | *CRP*-F | This paper | dCas9 targeting sequences | GGGGACTGTTGTGGGGTGGG |
| sequence-based reagent | *CRP*-R | This paper | dCas9 targeting sequences | GAAGCTCTGACACCTGCCCC |
| sequence-based reagent | *C/EBP-β*-F | This paper | Cas9 targeting sequences | GGGCGCCTGGGGGCCGCCAA |
| sequence-based reagent | *C/EBP-β*-R | This paper | Cas9 targeting sequences | GGCGGCGGCGGCGGCGGGGG |
| commercial assay or kit | EpiTect Bisulfite Kit | QIAGEN | catalog number: 59104 |  |
| commercial assay or kit | RNAiso Plus reagent | Takara | catalog number: 9108 |  |
| commercial assay or kit | Dual-Luciferase Reporter Assay System | Promega | catalog number: E1960 |  |
| chemical compound, drug | 5-Azacytidine | Sigma-Aldrich | catalog number: A2385 |  |
| chemical compound, drug | RG108 | Selleck Chemicals | catalog number: S2821 |  |
| chemical compound, drug | Blasticidine | Sigma-Aldrich | catalog number: 15205 |  |
| chemical compound, drug | puromycin | Corning | catalog number: 58-58-2 |  |
| chemical compound, drug | BAY11-7082 | Selleck Chemicals | catalog number: S2913 |  |
| chemical compound, drug | Stattic | Selleck Chemicals | catalog number: S7024 |  |
| chemical compound, drug | S31-201 | Selleck Chemicals | catalog number: S1155 |  |
| software, algorithm | Origin 8.0 | OriginLab |  |  |
| software, algorithm | BiQ_Analyzer | Bock *et al*, 2005, Bioinformatics, 21: 4067 |  |  |
| software, algorithm | Oligo 7.0 | Molecular Biology Insights |  |  |
| software, algorithm | Clustalx 1.81 | Larkin *et al*, 2007, Bioinformatics, 23: 2947 |  |  |
